# Production of an Attenuated Phenol-Soluble Modulin Variant Unique to the MRSA Clonal Complex 30 Increases Severity of Bloodstream Infection

**DOI:** 10.1371/journal.ppat.1004298

**Published:** 2014-08-21

**Authors:** Gordon Y. C. Cheung, Dorothee Kretschmer, Anthony C. Duong, Anthony J. Yeh, Trung V. Ho, Yan Chen, Hwang-Soo Joo, Barry N. Kreiswirth, Andreas Peschel, Michael Otto

**Affiliations:** 1 Pathogen Molecular Genetics Section, Laboratory of Human Bacterial Pathogenesis, National Institute of Allergy and Infectious Diseases, The National Institutes of Health, Bethesda, Maryland, United States of America; 2 Cellular and Molecular Microbiology Division, Interfaculty Institute of Microbiology and Infection Medicine, University of Tübingen, Tübingen, Germany; 3 Public Health Research Institute Tuberculosis Center, New Jersey Medical School, Rutgers University, Newark, New Jersey, United States of America; Columbia University, United States of America

## Abstract

Methicillin-resistant *Staphylococcus aureus* (MRSA) is a leading cause of morbidity and death. Phenol-soluble modulins (PSMs) are recently-discovered toxins with a key impact on the development of *Staphylococcus aureus* infections. Allelic variants of PSMs and their potential impact on pathogen success during infection have not yet been described. Here we show that the clonal complex (CC) 30 lineage, a major cause of hospital-associated sepsis and hematogenous complications, expresses an allelic variant of the PSMα3 peptide. We found that this variant, PSMα3N22Y, is characteristic of CC30 strains and has significantly reduced cytolytic and pro-inflammatory potential. Notably, CC30 strains showed reduced cytolytic and chemotactic potential toward human neutrophils, and increased hematogenous seeding in a bacteremia model, compared to strains in which the genome was altered to express non-CC30 PSMα3. Our findings describe a molecular mechanism contributing to attenuated pro-inflammatory potential in a main MRSA lineage. They suggest that reduced pathogen recognition via PSMs allows the bacteria to evade elimination by innate host defenses during bloodstream infections. Furthermore, they underscore the role of point mutations in key *S. aureus* toxin genes in that adaptation and the pivotal importance PSMs have in defining key *S. aureus* immune evasion and virulence mechanisms.

## Author Summary

Methicillin-resistant *Staphylococcus aureus* (MRSA) is a major cause of morbidity and mortality and a great concern for public health. The CC30 MRSA lineage is especially notorious for causing bloodstream infections with complications such as seeding into organs. In our study, we show that this lineage produces an attenuated form of a key *S. aureus* toxin with decreased pro-inflammatory features. Our results suggest that attenuation of this toxin allows the bacteria to evade recognition and subsequent elimination by host defenses, thereby increasing pathogen success during blood infection.

## Introduction

*Staphylococcus aureus* is a dangerous human pathogen that is responsible for thousands of deaths annually in the U. S. alone [1]. Virulence of *S. aureus* is due to a large repertoire of virulence factors, including immune evasion factors and aggressive cytolytic toxins [2]. *S. aureus* infections become particularly dangerous when they are caused by strains that are resistant to commonly used antibiotics. Methicillin-resistant *S. aureus* (MRSA) is of especially great concern, as identification of MRSA eliminates the therapeutic use of most beta-lactam antibiotics, which are antibiotics of first choice against pathogenic staphylococci. Many countries report high rates of methicillin resistance among hospital-associated infections caused by *S. aureus* [3]. In addition, community-associated strains of MRSA (CA-MRSA) have emerged over the last two decades that have the capacity to infect healthy individuals outside of hospital settings [4].

MRSA strains belonging to clonal complex (CC) 30 are a major cause of hospital-associated infections in the U. S., Europe, and elsewhere [5]–[7]. Infections with CC30 MRSA present predominantly as bloodstream infections with complications such as hematogenous seeding [8]. Historical methicillin-susceptible CC30 strains (phage type 80/81) caused serious, in part community-associated infections of the skin and lungs in addition to blood infections. In contrast to contemporary CC30 isolates, many historical phage type 80/81 clones had genes encoding the Panton-Valentine leukocidin (PVL) [7]. Furthermore, contemporary CC30 clones contain mutations in the global virulence regulator Agr (*agrC* gene, non-synonymous mutation, G55R) and the gene encoding α-toxin (*hla*, STOP mutation) [9]. The resulting overall lower expression of cytolytic toxins in contemporary compared to historic CC30 clones has been linked to the fact that contemporary CC30 clones predominantly cause hospital-associated infections [9].

PSMs are short, amphipathic, α-helical peptides with a major impact on *S. aureus* virulence [10], [11]. The PSMα peptides of *S. aureus* in particular cause lysis of a variety of cell types, including neutrophils (or polymorphonuclear leukocytes, PMNs), monocytes, erythrocytes, and osteoblasts [11]–[13]. The rather low target specificity of PSM-mediated cytolysis is due to the fact that lysis is believed to be receptor-independent [14], [15], which is reflected by the capacity of PSMs to lyse artificial vesicles [14]. In addition, PSMs have pro-inflammatory capacities that are receptor-dependent, leading for example to neutrophil chemotaxis and activation [11], [14]. Similar to other *S. aureus* toxins, these immune-stimulatory activities are observed at sublytic concentrations [14], [16]. PSMα3 is the by far most pro-inflammatory and cytolytic PSM of *S. aureus* [11]. Notably, the capacity of PSMα3 to elicit chemotaxis by neutrophils by far exceeds that of any other PSM of *S. aureus* [11].

Except for a variation in the PSM δ-toxin sequence (serine substitution for glycine at position 10 in some strains), whose effect on peptide function has not yet been analyzed, naturally occurring variants of PSM peptides have not yet been reported; and in general, the consequences of non-synonymous variations in *psm* genes are not understood. Here, we report an allelic variation in the PSMα3-encoding gene that is characteristic of CC30 strains and leads to significantly lower cytolytic and chemotactic activity, and increased hematogenous seeding in a bacteremia model. For the first time, our study describes an allelic variant of a *psm* gene that has key biological consequences and whose appearance is strongly correlated with a specific MRSA lineage. Furthermore, our findings reveal a molecular mechanism supporting the notion that MRSA strains such as those of the CC30 lineage evolved to cause specific infections, in which reduction of the expression of immune-stimulatory toxins allows to evade recognition and elimination by host defenses.

## Results

### CC30 strains contain a unique non-synonymous mutation in the *psm*α3 gene

**Figure 1:**
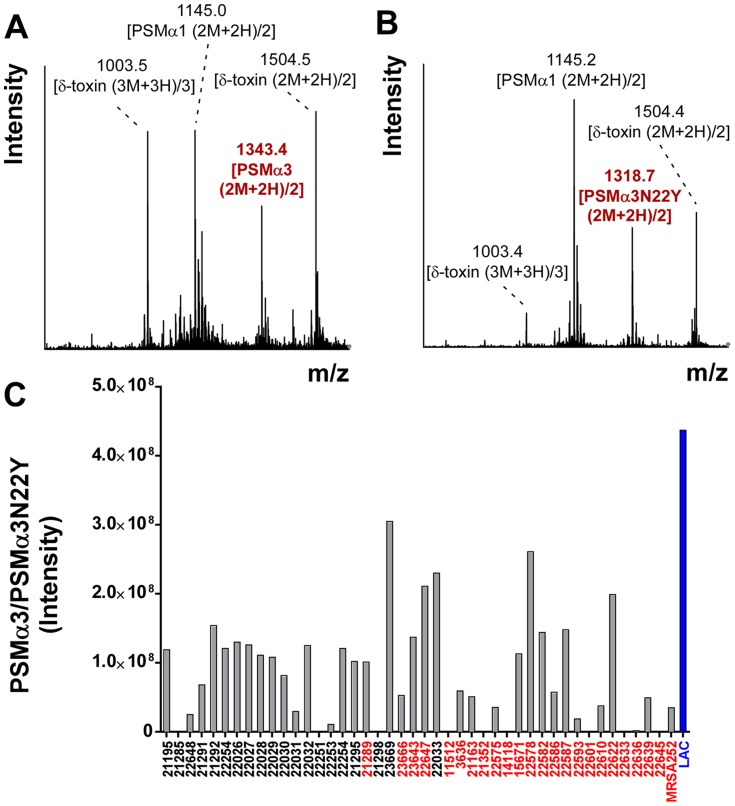
CC30 strains produce a variant of PSMα3. (A,B) Mass spectrogram of the region where PSMα3 elutes during RP-HPLC/MS of the culture filtrate of a non-CC30 strain (USA300) (A) or a CC30 strain (B), respectively. The peak corresponding to PSMα3 is marked in red. Other peaks are from co-eluting PSM peptides. (C) PSMα3N22Y production in a collection of historic and contemporary CC30 strains. Strains shown in red contain a mutation in *agrC* that is characteristic of contemporary CC30 strains. Production of PSMα3 in strain LAC (USA300) is shown as comparison.

When analyzing CC30 strains using an HPLC/MS-based PSM analysis method [17], we realized that CC30 strains lacked the m/z values typical for PSMα3, but instead produced a peptide with a changed mass (Fig. 1A,B). The mass of that peptide was 49 Da higher than that of PSMα3. Sequencing the *psm*α3 gene in selected CC30 strains and comparing to the published genome of the CC30 strain MRSA252 (EMRSA16) [18], we identified the cause for that difference as a non-synonymous mutation in the *psm*α3 gene in the codon coding for the C-terminal amino acid position (TAC instead of AAC, leading to an exchange of asparagine with tyrosine). Notably, we found the mass corresponding to the PSMα3N22Y peptide in all CC30 strains that we analyzed, containing contemporary and historic strains (Fig. 1C) [9], but never in any of the large number of *S. aureus* strains of different genetic backgrounds that we had analyzed over the last ∼5 years. Some CC30 strains did not produce any PSM peptide (9 among the 41 analyzed). Lack of PSM production is commonly found in about a quarter of strains in staphylococcal strain collections and due to non-functionality of the Agr system, which strictly regulates PSM production [19], [20]. Furthermore, a BLASTP search only detected the PSMα3N22Y peptide in the CC30 strain MRSA252, but not in any other sequenced *S. aureus* genome. Therefore, we conclude that the N22Y variant of PSMα3 is characteristic of the CC30 lineage.

### Production of α-toxin, but not PSM production and Agr functionality, is significantly different in contemporary and historic CC30 strains

**Figure 2:**
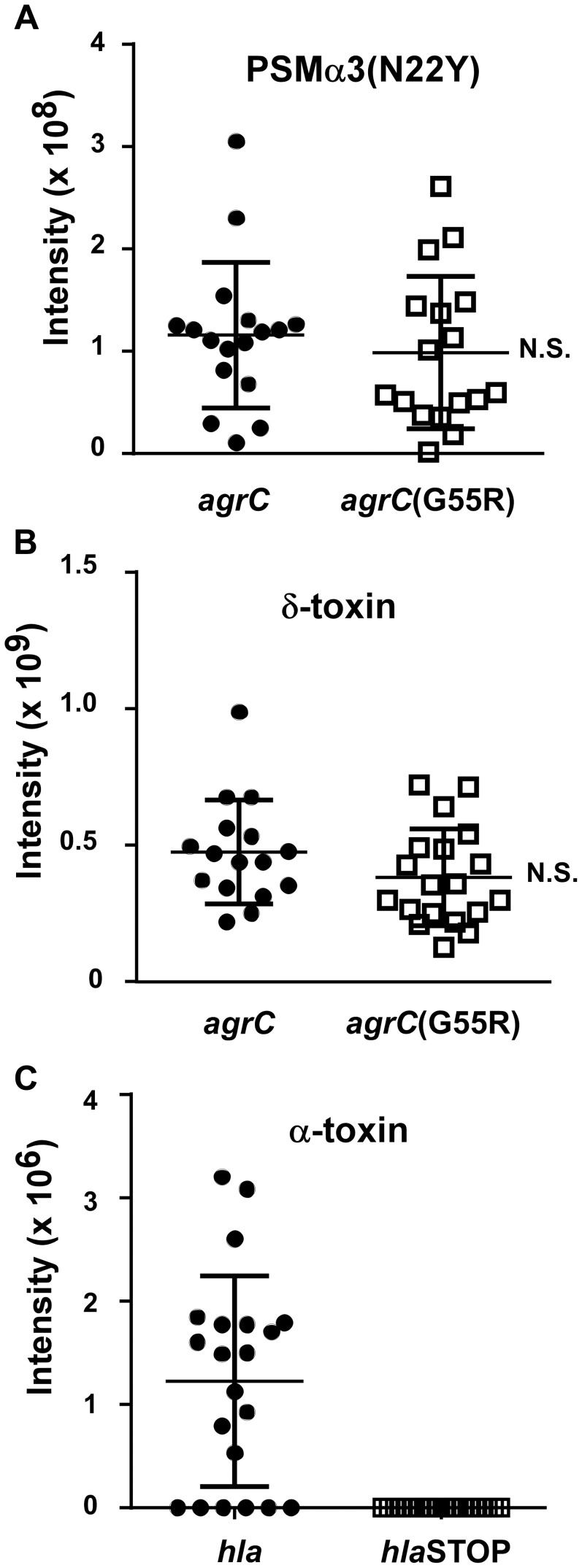
PSM and α-toxin production in historic and contemporary CC30 MRSA strains. (A,B) Production of PSMα3 and δ-toxin in culture filtrates of a CC30 strain collection (see Fig. 1C). PSMs were measured by HPLC/MS. (C) Production of α-toxin, which was measured by Western blots and densitometry of α-toxin bands in the same culture filtrates. N.S., not significant. For the comparison of α-toxin production, no significance could be computed, as all contemporary strains with *hla* mutations completely lacked α-toxin production. One strain in the collection contains only the *agrC*, but not the *hla* mutation, and was grouped according to presence/absence of those mutations.

Contemporary CC30 MRSA strains contain two key mutations, one in the *hla* (α-toxin) and one in the *agrC* gene [9]. When introduced in a historic CC30 strain (strain 22030), both mutations caused a significant reduction in virulence in a mouse infection model; and the *agrC* mutation led to reduced production of RNAIII [9], the intracellular effector molecule of the Agr system [21]. However, we did not detect a significant difference in the average PSMα3N22Y production comparing historic and contemporary CC30 strains from that previous study [9] (Fig. 2). Given that PSM production is a direct readout of Agr functionality [19], our present results indicate that the *agrC* mutation in contemporary CC30 strains does not cause a significant reduction of Agr functionality in average in the contemporary CC30 strain population. We confirmed the results obtained with PSMα3 by analyzing production of δ-toxin, which is the translational product of RNAIII. In both PSMα3 and δ-toxin analyses, contemporary CC30 strains only showed a slightly reduced average production (PSMα3, 0.99×10^7^ versus 1.16×10^8^ intensity units; δ-toxin 3.18×10^8^ versus 3.81×10^8^); both differences were not significant (Fig. 2). In contrast, production of α-toxin as measured by densitometry of Western blot signals was strongly different between the historical CC30 strains and the contemporary strains bearing a stop codon mutation in the α-toxin gene (Fig. 2). As expected, no α-toxin could be detected in culture filtrates of the latter. These findings attribute a greatly more important impact of the *hla* than the *agrC* mutation to causing overall lower aggressive virulence of contemporary as compared to historic CC30 strains.

### PSMα3N22Y has decreased chemotactic activity

**Figure 3:**
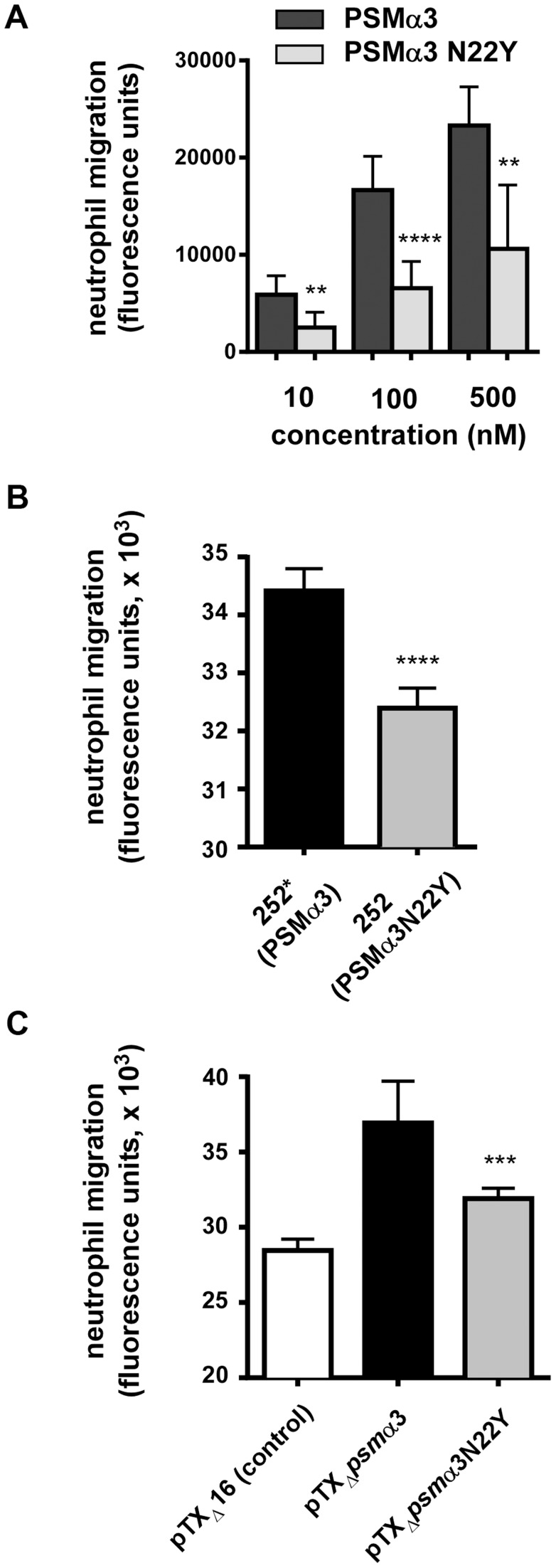
PSMα3N22Y has decreased chemotactic activity toward human neutrophils. (A) Chemotaxis of human neutrophils (neutrophil migration) was measured using a transwell system with synthetic peptides at the indicated concentrations. Neutrophils were from three donors and the measurements were performed in quadruplicate. **, p<0.01; ****, p<0.0001. (B) Chemotaxis of human neutrophils tested with culture filtrates (diluted 1∶100) of strains MRSA252 (252, expressing PSMα3N22Y) and 252* (MRSA252 altered to express PSMα3); ****, p<0.0001. Data are from two donors with triplicate measurements. (C) Chemotaxis of human neutrophils tested with culture filtrates (diluted 1∶100) of strains expressing PSMα3 or PSMα3N22Y constitutively from plasmids; pTX_Δ_16 is the empty control plasmid. ***, p<0.001 (versus pTX_Δ_ *psm*α3). Data are from two donors with triplicate measurements.

Neutrophil chemotaxis is one of the most important pro-inflammatory activities exerted by PSMs via their interaction with formyl peptide receptors (FPRs) [10], [14]. PSMs activate FPRs at nanomolar amounts, with activation of FPR2 (also termed FPRL1) being ∼10 times stronger than that of FPR1 and FPR3 [14]. We analyzed the capacities of PSMα3 to cause neutrophil chemotaxis in comparison to PSMα3N22Y. PSMα3 showed a capacity to cause neutrophil chemotaxis that was about twice higher than that caused by PSMα3N22Y at all tested concentrations (Fig. 3A).

To test the impact of the *psm*α3 gene mutation in CC30 strains on chemotactic activity in the natural strain background, we changed the corresponding, last codon in the *psm*α3 coding sequence in the genome of strain MRSA252 by an allelic replacement-based strategy to the sequence found in other *S. aureus* strains (from TAC to AAC, tyrosine to asparagine codon). Furthermore, we analyzed the impact of PSMα3 and PSMα3N22Y expressed constitutively from a plasmid in a PSM-negative strain background (strain USA300 with all *psm* genes deleted) [22]. PSMα3N22Y and PSMα3 expression was verified by HPLC/MS and found to be highly similar in the respective strain pairs (Supplemental Fig. S1). Chemotaxis of human neutrophils was significantly higher with the MRSA252 strain that was altered to express non-CC30 PSMα3 (strain 252*) compared to the wild-type strain MRSA252 expressing PSMα3N22Y (Fig. 3B). Similarly, chemotaxis was significantly higher in the PSM-free strain expressing PSMα3 from a plasmid compared to the isogenic strain expressing PSMα3N22Y (Fig. 3C). These results demonstrate that PSMα3N22Y causes decreased chemotaxis of human neutrophils compared to PSMα3, also when expressed in its natural strain background.

### Diminished activation of FPR1 plays contributes significantly to the differences in pro-inflammatory activities of PSMα3N22Y versus PSMα

**Figure 4:**
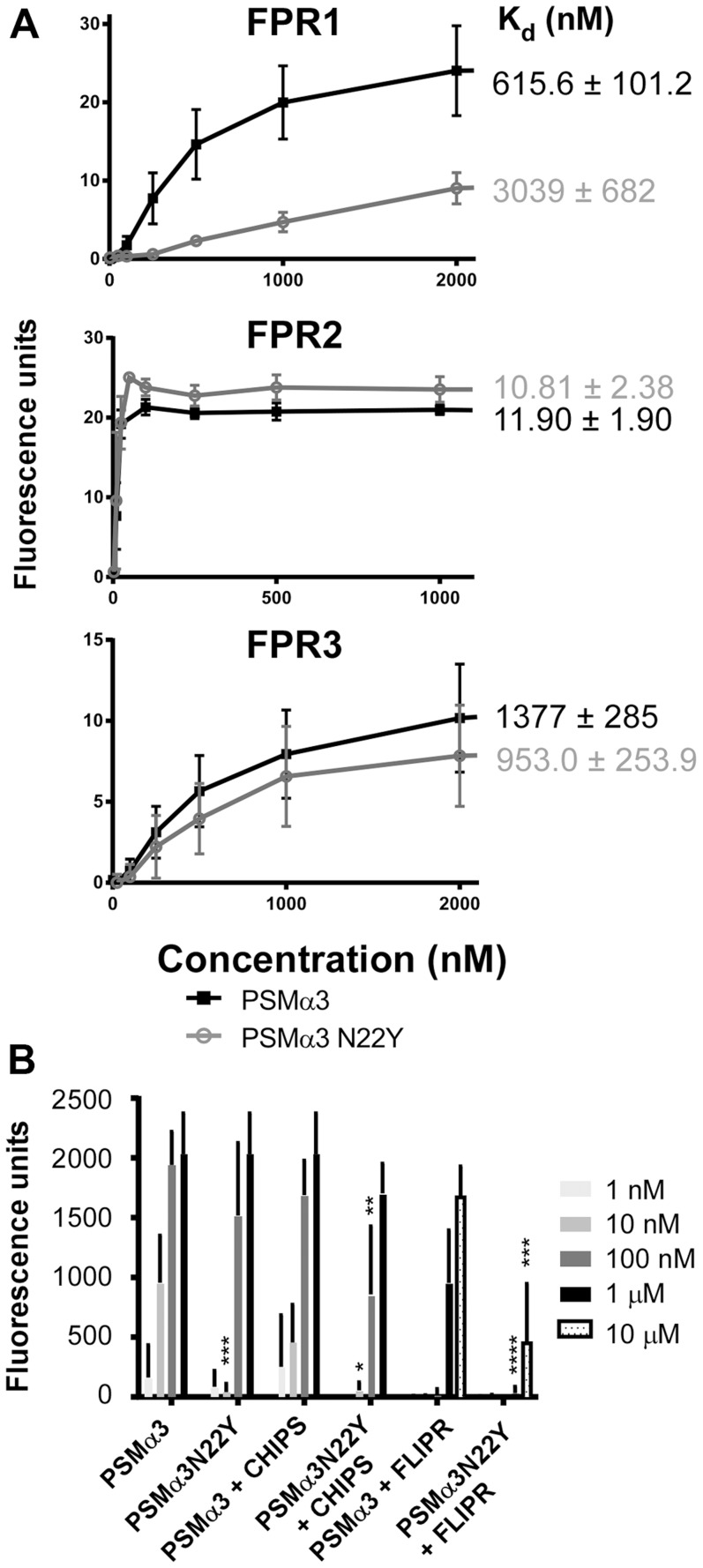
PSMα3N22Y shows decreased activation of FPR1. (A) Differential activation of FPRs by PSMα3 versus PSMα3N22Y was measured using synthetic peptides and Ca^2+^ flux observed in HL60 cells stably transfected with the corresponding FPR genes as readout, using three biological and two technical replicates. The calculated K_d_ values are shown on the right. The obtained curves were significantly different for PSMα3 versus PSMα3N22Y only in the case of FPR1. (B) Ca^2+^ flux in human neutrophils upon addition of PSMα3 or PSMα3N22Y and inhibition by FLIPr (FPR2 inhibitor) or CHIPS (FPR1 inhibitor). Neutrophils were from three donors and the measurements were performed in quadruplicate. *, p<0.05; **, p<0.01; ***, p<0.001; ****, p<0.0001 (comparing values obtained with PSMα3 versus corresponding values obtained with PSMα3N22Y).

Next, we analyzed Ca^2+^ flux as a general measure to test for neutrophil activation, as used in previous studies [14]. HL60 cells stably transfected with different FPR receptor genes [14] were used to determine which FPR receptors are differentially activated by PSMα3 versus PSMα3N22Y (Fig. 4A). Somewhat surprisingly, only FPR1 was activated differentially by the two peptides in this assay, but not FPR2 and FPR3. As expected, both peptides activated FPR2 much stronger than FPR1 and FPR3, but we detected no difference in the FPR2-activating potential between the two peptides. Using a one site-binding model, we obtained K_d_ values for FPR1 of 615.6±101.2 nM (PSMα3) versus 3039±682 nM (PSMα3N22Y), which represents a significant difference with a factor of ∼5. In contrast, K_d_ values were not significantly different for FPR2 (PSMα3, 11.90±1.90 nM; PSMα3N22Y, 10.81±2.38 nM) and FPR3 (PSMα3, 1377±285 nM; PSMα3N22Y, 953.0±253.9). However, in human neutrophil chemotaxis experiments using specific inhibitors of FPR1 (chemotaxis inhibitory protein of *S. aureus*, CHIPS) and FPR2 (FPRL1 inhibitory protein FLIPr) [23], [24], we found that both FPR1 and FPR2 appear to be responsible for the observed differences between PSMα3 and PSMα3N22Y (Fig. 4B). At a peptide concentration of 100 nM, we observed a difference of ∼factor 2 in chemotactic activity comparing the two peptides when FPR1 recognition was inhibited by CHIPS, likely attributable to FPR2-mediated recognition. Owing to the overall ∼10 times lower capacity to stimulate FPR receptors other than FPR2, we observed no chemotaxis at that peptide concentration with addition of the FPR2 blocking agent FLIPr. However, at a 10 times higher peptide concentration of 1 µM, there was a very strong difference in chemotactic potential when FPR2-mediated recognition was blocked (Fig. 4B), which is in agreement with the results of the transfected HL60 experiments and according to our results mainly attributable to differences in activation of FPR1. Together, these results indicate that the differences in the pro-inflammatory potentials of PSMα3 and PSMα3N22Y are due to a considerable extent to differential activation of FPR1, while the overall stronger activation of FPR2 does not differ that much between the two peptides.

### PSMα3N22Y has reduced cytolytic activity toward human neutrophils and erythrocytes compared to PSMα3

PSMα peptides are strongly cytolytic to a variety of cell types, which is believed to be a major determinant of their impact on disease progression observed in several manifestations of *S. aureus* disease, in particular infections of the skin [10]. PSM-mediated cytolysis is assumed to occur independently of receptor interaction by promoting disintegration of eukaryotic plasma membranes [14], [15], [25].

**Figure 5:**
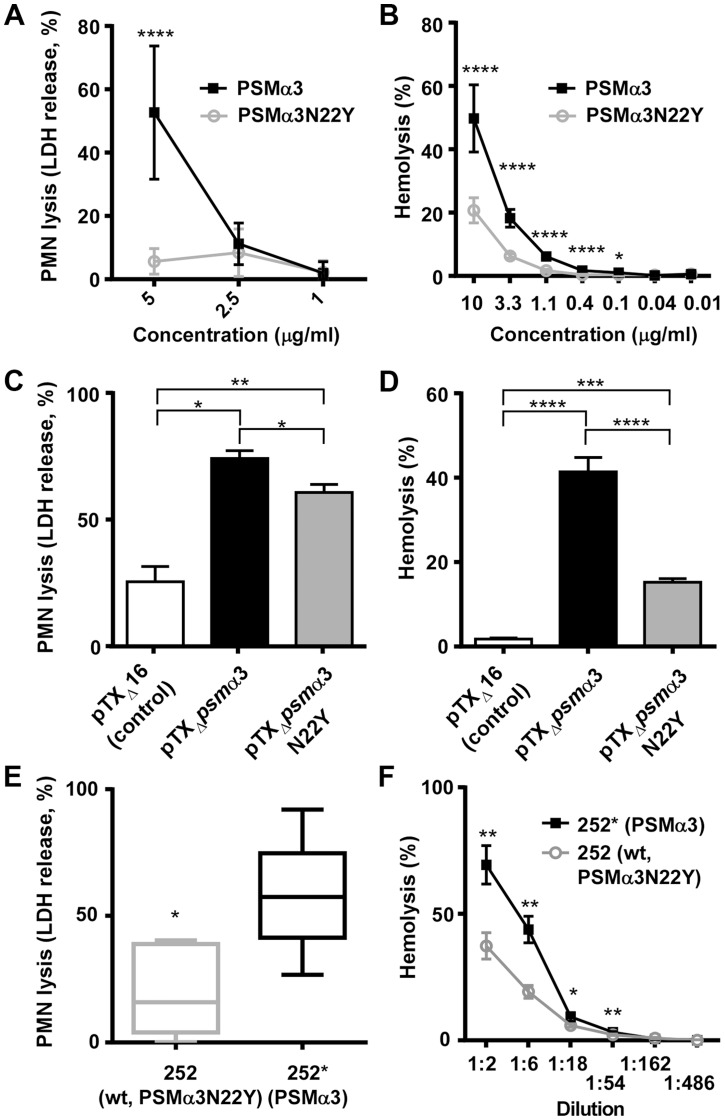
PSMα3N22Y has diminished cytolytic activities. (A) PMN lysis of synthetic PSMα3N22Y versus PSMα3. Data are from 2 donors, measured in triplicate each after incubation at 37°C for 1 h. At concentrations of 10 µg/ml or higher, both peptides completely lysed the neutrophils. (B) Hemolysis of synthetic PSMα3N22Y versus PSMα3. Data are from 2 donors, measured in triplicate, after incubation at 37°C for 1 h. (C, D) PMN lysis and hemolysis, respectively, of culture filtrates from PSMα3N22Y- versus PSMα3-producing strains when expressed on plasmids. pTX_Δ_16 is the empty control vector. The background strain in all constructs is a PSM-free USA300 strain (LACΔ*psm*αΔ*psm*βΔ*hld*). Culture filtrates were diluted 1∶20 for the PMN lysis and 1∶2 for the hemolysis measurements. PMN lysis results are from two and hemolysis data from one donor, with three replicates measured each. (E) PMN lysis of culture filtrates from MRSA252 (252) versus 252*, in which the *psm*α3 gene (expressing PSMα3N22Y) on the genome was changed to express PSMα3 as present in all non-CC30 strains. The graph shows the results from six different donors. Culture filtrates were diluted 1∶10. *, p<0.05 (paired t-test). (F) Hemolysis of culture filtrates from the same strains as in (E) at different dilutions. (A–F), *, p<0.05; **, p<0.01; ***, p<0.001; ****, p<0.0001.

To compare the cytolytic capacities of PSMα3 and PSMα3N22Y, we first analyzed the degree to which synthetic peptides lysed human erythrocytes and neutrophils. PSMα3 showed significantly higher cytolytic capacities toward human neutrophils and erythrocytes compared to PSMα3N22Y (Fig. 5A,B). Then, we analyzed the impact of PSMα3 and PSMα3N22Y on cytolysis when expressed from plasmids and the genome (see above). In accordance with the results achieved with the synthetic peptides, culture filtrates of the PSMα3-expressing strain had significantly higher capacities to lyse human erythrocytes and neutrophils (Fig. 5C–F). These findings show that the CC30 *psm*α3 gene mutation leads to significantly decreased cytolytic activity toward human neutrophils and erythrocytes, including in its original strain background.

### The *psm*α3 non-synonymous mutation in CC30 isolates leads to increased virulence during bacteremia

**Figure 6:**
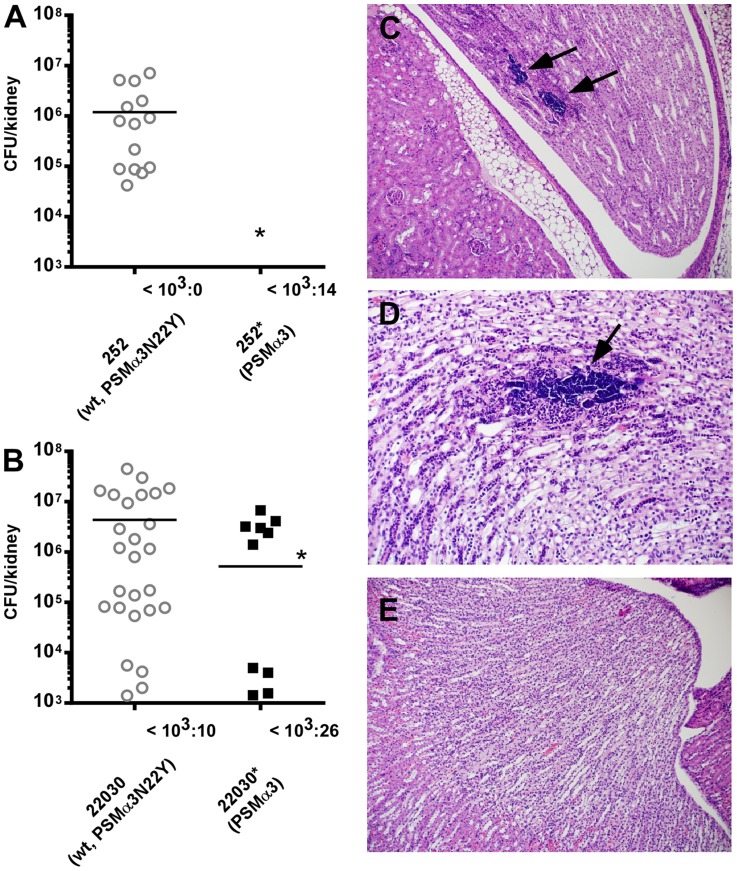
The PSMα3 variation significantly impacts virulence of CC30 MRSA in experimental blood infection. (A) The MRSA252 strain (252, expressing PSMα3N22Y) was compared to a strain (252*) in which the *psm*α3 gene was changed to express non-CC30 PSMα3 in a mouse model of blood infection. Hematogenous seeding into the kidneys was measured 4 days after infecting mice via the tail vein with 1×10^7^ CFU. Results from kidneys with considerable bacterial numbers (>10^3^ CFU) are shown in the graph; the number of kidneys with lower or no bacteria is shown at the bottom. Analysis of significance was performed with the numbers obtained from all kidneys; *, p<0.05. Number of mice used per strain: 10. Two counts per analyzed kidney were performed. (B) Experiment as in (A) with strain 22030 expressing PSMα3N22Y and the isogenic 22030* strain expressing PSMα3. Number of mice used per strain: 20; two counts per analyzed kidney are included; inoculum: 1×10^6^. *, p<0.05. (C, D) Tubulointerstitial nephritis with large colonies of bacteria (arrows) in kidneys infected with strain 252, magnification 100× (C) and 200× (D). (E) Absence of signs of infection in kidneys infected with strain 252* (magnification 100×).

The CC30 MRSA lineage is a leading cause of sepsis and subsequent hematogenous complications in hospitals. Therefore, we used a mouse bacteremia (renal abscess) model with assessment of seeding into and abscess formation in the kidneys to evaluate the impact of the *psm*α3 mutation on CC30 pathogenic success. Both the contemporary CC30 MRSA strain 252 and the historic strain 22030 showed significantly increased seeding into kidneys and kidney abscess formation compared to the respective isogenic mutants in which the *psm*α3 gene was altered to the non-CC30 form (Fig. 6A, B). Considerable bacterial numbers (>1000/kidney) were found in 70% of kidneys in mice infected with MRSA252, while never in kidneys infected with the isogenic mutant expressing the non-CC30 form of PSMα3 (Fig. 6A). Similar results were found for the 22030 strain pair (65% versus 20%) (Fig. 6B). Histological analyses of several kidneys from mice infected with MRSA252 showed clear signs of infection: they had developed tubulointerstitial nephritis that was associated with colonies of cocci (Fig. 6 C, D). This was not seen in the kidneys of mice infected with the isogenic strain expressing the non-CC30 form of PSMα3 (Fig. 6E). These results indicate that the specific features of the CC30 mutant of PSMα3 (PSMα3N22Y) result in an increased capacity to cause sepsis and subsequent complications. This effect appeared to be stronger in the contemporary clone MRSA252 than in the historic strain 22030, possibly because the latter strongly expresses a series of other pro-inflammatory toxins that to a certain extent overshadow the effect of the PSMα3 mutation.

PSMs of the α-type are known to have a strong impact on acute skin infections [11], [26]. This is believed to be due primarily to their cytolytic activities, which they exert for example on neutrophils after phagocytosis [27]. We found that the CC30 form of PSMα3 (PSMα3N22Y) in strains MRSA252 and 22030 did not cause increased virulence in skin infections as measured by abscess sizes. Rather, abscess sizes were in general slightly larger in the MRSA252 strain expressing non-CC30 PSMα3 compared to the MRSA252 wild-type strain, but differences were not significant. No differences were detectable with the 22030 strain pair (Supplemental Fig. S2). This indicates that in contrast to bacteremia, the altered features of the CC30-type PSMα3 (PSMα3N22Y) do not significantly impact the outcome of skin infections.

## Discussion

In the present study, we report an allelic variant in a *psm* gene that is characteristic for a specific *S. aureus* lineage, namely CC30, and shows reduced immune-stimulatory and cytolytic activities. Our results indicate that the increased capacity of CC30 strains to cause hematogenous complications is at least in part due to the production of this attenuated form of PSMα3. Furthermore, the strongly different impact of that form of PSMα3 on bacteremia versus skin infection underscores that the two main features of PSMs, i.e. cytolytic and pro-inflammatory capacity, may have a strongly different influence on the development of distinct disease types.

Given that a reduction in cytolytic activity can hardly explain increased virulence and hematogenous seeding, the differences that we detected in the bacteremia model are very likely due to the attenuated pro-inflammatory features of the attenuated form of PSMα3 in CC30 strains. These findings are in accordance with the notion that the innate immune system makes use of *S. aureus* toxins for pathogen recognition and evading that recognition is of benefit for bacterial survival in specific types of disease [28]. This notion is founded on several previous observations: first, *S. aureus* produces a series of molecules such as CHIPS or FLIPr that block toxin recognition by receptors on innate host defense cells, including the FPRs that recognize PSMs [14], [23], [24]; second, it was found that the pro-inflammatory properties of PVL [16], [29] enhanced clearing of pneumonia and anti-PVL antibodies led to increased severity of infection [30]–[32]. Finally, persistent bacteremia has been reported to be associated with Agr dysfunction [33]. While the latter has been explained by an increased fitness cost associated with the production of RNAIII [34], this correlation may also be explained by the lower expression of pro-inflammatory molecules in *agr* mutants (such as PSMs and other toxins). All these observations indicate that toxin production is a double-edged sword for the bacteria: toxins not only serve to eliminate immune cells but also trigger the launch of host defenses, representing a sort of pathogen-associated molecular pattern. Importantly, our study is the first to provide an example in which a staphylococcal toxin variant appears to have evolved to circumvent pathogen recognition.

The *psm*α3 allelic variant is present in all CC30 strains, including historic and contemporary strains. Our findings therefore do not answer the much-debated question which specific mutations are linked to the fact that the latter are predominantly restricted to the hospital setting [7], [9]. However, our findings suggest that the *hla* and *agrC* mutations in contemporary CC30 clones may add to immune evasion by lowering pathogen recognition in a similar fashion as the PSMα3 attenuation, because they also result in an attenuation of the expression of pro-inflammatory toxins. These mutations, which are believed to make contemporary strains of CC30 better adapted to long-term colonization and persistence in the human host, may thus not decrease virulence in a general fashion as has been suggested [9]. They may rather shift the disease spectrum to types of infection in which the production of aggressive toxins that alert the immune system is counterproductive for the establishment and progression of infection. According to our results, the *hla* mutation plays a much greater role in that adaptation of contemporary CC30 clones to hospital-associated infections than the *agrC* mutation, Altogether, these findings attribute a key role to point mutations in major *S. aureus* toxins for virulence and correlation with specific disease types.

Results that we previously obtained using an alanine exchange peptide library screen of PSMα3 indicated that the C-terminal amino acids are critical for interaction with FPR2 [35]. In general, the difference in receptor interaction between PSMα3 and PSMα3N22Y as determined in the present study supports this concept. However, exchange of the C-terminal asparagine with tyrosine (CC30 PSMα3N22Y) leads to a different change in receptor interaction as compared to an exchange with alanine (PSMα3N22A), inasmuch as the former more strongly affects interaction with FPR1. This indicates that the molecular details of PSM – FPR interaction differ between the different subtypes of FPRs.

In conclusion, we here report an allelic variation of a key member of the PSM toxin family that significantly impacts in-vitro virulence phenotypes and in-vivo virulence of strains of the CC30 lineage. Our findings lend support to the general idea that mutations leading to lower pro-inflammatory potential are linked to the involvement in specific types of disease.

## Materials and Methods

### Ethics statement

The used animal protocol (LHBP1E) was reviewed and approved by the Animal Use Committee at NIAID, NIH, according to the animal welfare act of the United States (7 U.S.C. 2131 et. seq.). Human neutrophils were isolated from venous blood of healthy volunteers in accordance with protocols approved by the Institutional Review Board for Human Subjects, NIAID, and the University of Tübingen, Germany. Informed written consent was obtained from all volunteers.

### Bacterial strains and culture conditions

The bacterial strains used in this study are contemporary and historic CC30 strains as published in a previous study [9]. MRSA252 is a widespread contemporary CC30 isolate and a frequent cause of hospital-associated infections [18]. LAC is a CA-MRSA strain of the pulsed-field type USA300. Strains were grown in tryptic soy broth (TSB) with addition of tetracycline at 12.5 µg/ml when appropriate. Culture filtrates for all experiments were obtained from 50-ml cultures grown for 18 h in 100-ml flasks with shaking at 180 rpm. They were filtered with PES filters (0.2 µm pore size, Millipore) and used fresh or stored at −20°C for further use. Only for the analysis of the CC30 strain collection (Fig. 1C), strains were grown as 1-ml cultures in 5-ml tubes, with all other conditions being the same.

### Peptides

Synthetic PSM peptides were obtained from commercial vendors at a purity of >95%. They all carry the N-terminal N-formyl methionine as common for PSMs, which are secreted into the medium without a signal peptide [36]. Peptide stock solutions were made in dimethyl sulfoxide at 10 mg/ml, which were diluted in RPMI 1640 (Gibco) for PMN or Dulbecco's phosphate-buffered saline (DPBS) (Sigma) for hemolysis assays.

### Allelic replacement and plasmid construction

Allelic replacement was performed as previously described [11] using the plasmid pKOR1-based method [37] to change the *psm*α3 gene in MRSA252 and 22030 (encoding PSMα3N22Y) to non-CC30 *psm*α3. Template DNA was from strain LAC (USA300). Oligonucleotides used are shown in Table 1. Plasmids for PSM expression are based on plasmid pTX_Δ_, which is a derivative of the xylose-inducible plasmid pTX15 [38], in which inducible was changed to constitutive expression by deletion of the *xylR* repressor gene [11]. Oligonucleotides for construction of the pTX_Δ_ *psm*α3N22Y plasmid were used as reported [11] to amplify the *psm*α3 gene from genomic DNA of strain MRSA252. Fidelity of the replacement was ascertained by DNA sequencing of the *psm*α operon and HPLC/MS of PSMs showing the exchanged mass of PSMα3 versus PSMα3N22Y. Additionally, production of PSMs was verified in the culture filtrates and cultures of strains used for all experiments prior to performing the experiment, to ensure that PSMα3 and PSMα3N22Y production was at the same level and no *agr* or other mutation impacting PSM levels had occurred (see Supplemental Fig. S1).

**Table 1:** Oligonucleotides used in this study.

Name	Sequence (5′-3′)
For allelic replacement (change of MRSA252 or 22030 *psm*α3 encoding PSMα3N22Y to *psm*α3 encoding PSMα3)	
a3vFw	GGGGACAAGTTTGTACAAAAAAGCAGGCTATTAGCAGAACGCCAAGACG
a3vRv	GGGGACCACTTTGTACAAGAAAGCTGGGTCATGTTGACCATGAATACCG
a3vMutFw	GATCGTTAATGTTTGAGATTAGTTGTTACCTAAAAATT
a3vMutRv	TTTACTTGGTAAATTTTTAGGTAACAACTAATCTCAAA

### Measurement of PSM production by high-pressure liquid chromatography/mass spectrometry (HPLC/MS)

PSM concentrations in culture filtrates grown for 16 h in TSB were measured using HPLC/MS as described [17].

### Measurement of α-toxin by western blot

Staphylococcal strains were grown in TSB at 37°C overnight. 10 µl of these cultures were inoculated into 1 ml of TSB and grown for 8 h. Culture supernatants were loaded onto 15% SDS-PAGE gels and run at 150 V for 1 h. Proteins in the gels were blotted on nitrocellulose membranes using an iBlot Western blotting system (Life Technologies, Grand Island, NY). Blotted membranes were incubated with Odyssey blocking buffer (LI-COR, Lincoln, NE) for 1 h at room temperature. Anti-staphylococcal α-toxin rabbit serum (1∶2,000, Sigma-Aldrich, St. Louis, MO) was added to the blocking buffer and incubated for another hour at room temperature. Membranes were washed five times with washing buffer (Tris-buffered saline containing 0.1% Tween-20, pH 7.4) and incubated with 1∶10,000 diluted Cy5-labeled goat anti-rabbit IgG (Life Technologies, Grand Island, NY) in Odyssey blocking buffer in dark for 1 h at room temperature. Membranes were washed five times with the washing buffer and scanned with a Typhoon TRIO+ Variable Mode Imager (GE Healthcare, Piscataway, NJ). The amount of α-toxin in the scanned image was quantified using ImageQuant TL software (GE Healthcare, Piscataway, NJ).

### Measurement of neutrophil chemotaxis, lysis, and calcium flux

Human neutrophils were isolated from venous blood of healthy donors as described [24], [39]. Neutrophil chemotaxis and Ca^2+^ flux were determined as previously described [11], [24]. Briefly, neutrophils were subjected to a brief hypotonic shock with pyrogen-free water, washed, and suspended at 5×10^6^ cells/ml. Chemotaxis of neutrophils was determined using a transwell system (Costar) with analysis of neutrophil migration using fluorescence labeling. Calcium fluxes were monitored as a surrogate marker for chemotaxis since it can be measured more robustly and accurately than chemotaxis. For measurement of Ca^2+^ fluxes, 5×10^6^ neutrophils/ml were labeled with a fluorescent dye and analyzed with a FACScalibur (Becton Dickinson) as described [24]. For measuring the influence of CHIPS or FLIPr, 1×10^6^ cells/ml were pre-incubated with CHIPS or FLIPr at final concentrations of 1.4 µg/ml or 0.5 µg/ml, respectively, for 20 min at room temperature under agitation. The CHIPS and FLIPr proteins (a kind gift from K. van Kessel) were prepared as described [23], [24]. Measurements of 2,000 events were performed and Ca^2+^ flux was expressed as relative fluorescence corrected for buffer controls. For the measurement of neutrophil lysis, synthetic PSMs or (diluted) culture filtrates were added to wells of a 96-well tissue culture plate containing 10^6^ PMNs and plates were incubated at 37°C. At the desired times, PMN lysis was determined by release of lactate dehydrogenase (LDH) (Cytotoxicity Detection Kit, Roche Applied Sciences).

### Hemolysis assays

Hemolysis assays were performed according to Wang et al. [11] Briefly, whole blood from humans was washed twice with ice-cold DPBS. The final percentage of blood was 2% (v/v) in ice-cold DPBS. Equal volumes of diluted synthetic PSMs or dilutions of culture filtrates were incubated with the 2% erythrocyte suspension in a total volume of 200 µl in 96 round-bottomed plates. After incubation at 37°C for 1 h, the plates were centrifuged at 233×*g* at 4°C for 5 min, supernatants were collected, and the optical density was measured at 540 nm.

### Mouse model of skin infection

The mouse skin infection model was performed as described [11]. Briefly, six to eight week-old female Crl∶SKH1-hrBR hairless mice (Charles River Laboratories) were injected subcutaneously with ∼4×10^7^ CFU of *S. aureus* strains 252 or 252*, or ∼5×10^6^ CFU of *S. aureus* strains 22030 or 22030* in 50 µl of PBS in the left flank of the mouse. The length (L) and width (W) of the abscess or lesion caused by the bacterial infection was measured with an electronic caliper daily for 14 d post infection and calculated using the formula L×W. Typically, strain MRSA252 caused closed abscesses and strain 22030 open lesions. All animals were euthanized after completion of the entire procedure. All mouse experiments were performed blinded at the animal care facility of the NIAID, Building 33, in compliance with the guidelines of the NIAID/NIH Institutional Animal Care and Use Committee.

### Mouse bacteremia (renal abscess) model

For the renal abscess model, *S. aureus* strains were inoculated from a pre-culture and grown to mid-exponential growth phase (∼2 h), harvested, washed, and diluted with sterile PBS. Six to eight week-old female CD-1 mice (Charles River Laboratories) were infected with 50 µl of bacteria in PBS via the tail vein. Mice received ∼1×10^7^ CFU of *S. aureus* strains 252 or 252*, or ∼1×10^6^ CFU of *S. aureus* strains 22030 or 22030*. Four days post infection terminal cardiac bleeds were performed. There were no bacteria found in the blood. All mice were euthanized by CO_2_ inhalation and kidneys were collected. One kidney of each mouse was placed into a 2-ml tube containing 1 ml of sterile PBS with 500 mg of 2 mm borosilicate glass beads (Sigma). The kidney was homogenized in a Fast Prep bead beater (Thermo Savant) at 6 m/s for 20 s. The homogenates were diluted in PBS, plated onto TSB plates, and incubated overnight at 37°C for CFU counting. The other kidney was placed in 10% formalin (Sigma) for subsequent histopathological examination.

### Statistics

Statistical analysis was performed using Graph Pad Prism version 6.02. For the comparison of two groups, t-tests were used (unpaired unless otherwise noted), for three or more, 1-way or 2-way ANOVA, as appropriate. All error bars depict the standard deviation.

## Supporting Information

Figure S1 PSMα3/PSMα3N22Y production in MRSA252 and 22030 wild-type and genetically altered strains, and in PSM-free USA300 strains, in which the peptides were expressed from a plasmid. Data are from three independent cultures. Analysis was by HPLC/MS. The asterisk marks the strains in which the CC30 *psm*α3 gene was altered to express non-CC30 PSMα3.
(TIF)

Figure S2 Mouse model of skin infection. Female Crl∶SKH1-hrBR hairless mice were injected subcutaneously with ∼4×10^7^ CFU of *S. aureus* strains 252 or 252* (number of mice, 25 per group), or ∼5×10^6^ CFU of *S. aureus* strains 22030 or 22030* (number of mice, 15 per group) in 50 µl of PBS in the left flank of the mouse. The length (L) and width (W) of the abscess or lesion caused by the bacterial infection was measured with an electronic caliper daily for 14 d post infection and calculated using the formula L×W. Typically, strain MRSA 252 caused closed abscesses and strain 22030 open lesions.
(TIF)
