# Supplementary figures and images for: Production of an Attenuated Phenol-Soluble Modulin Variant Unique to the MRSA Clonal Complex 30 Increases Severity of Bloodstream Infection
Source: PLoS Pathog. 2014 Aug 21;10(8):e1004298. doi: 10.1371/journal.ppat.1004298 (PMC4140855; doi:10.1371/journal.ppat.1004298)

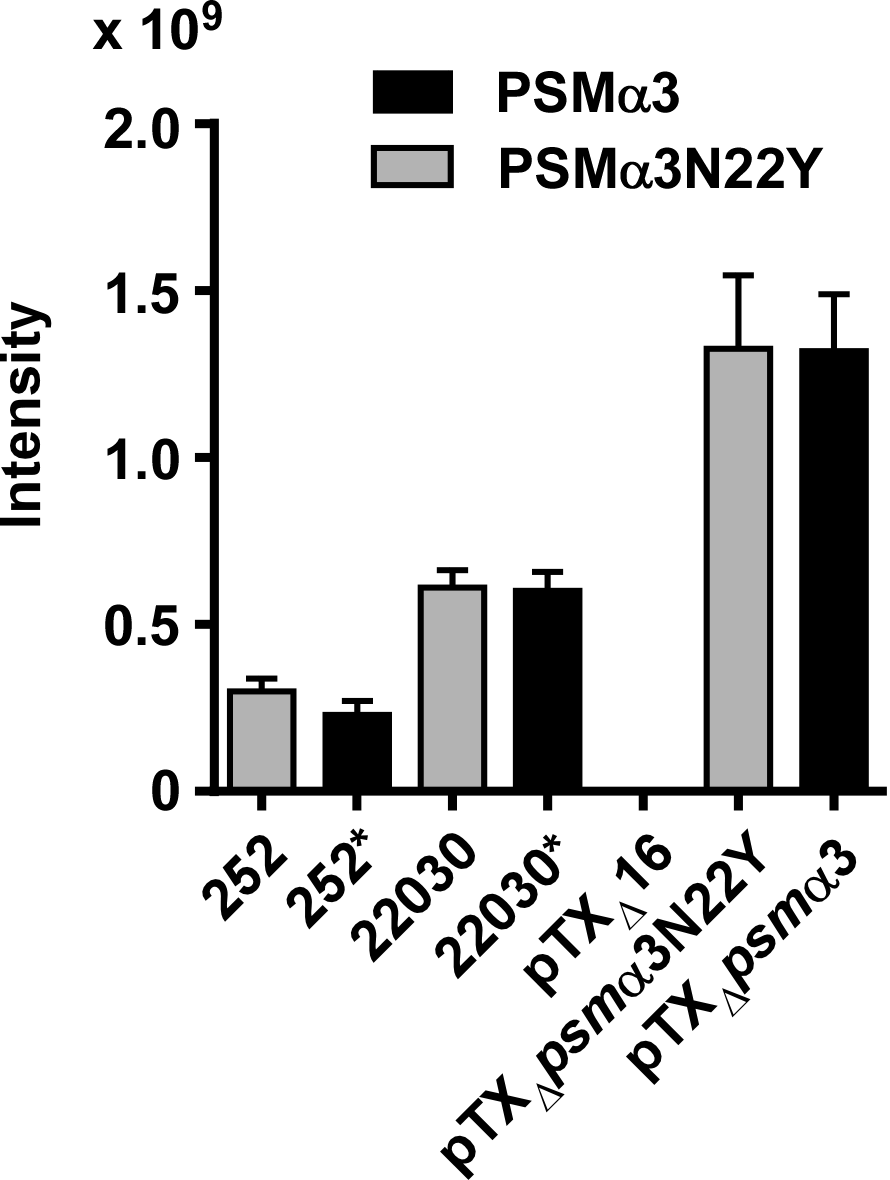

Supplement: Figure S1 — PSMα3/PSMα3N22Y production in MRSA252 and 22030 wild-type and genetically altered strains, and in PSM-free USA300 strains, in which the peptides were expressed from a plasmid. Data are from three independent cultures. Analysis was by HPLC/MS. The asterisk marks the strains in which the CC30 psmα3 gene was altered to express non-CC30 PSMα3. (TIF) [file ppat.1004298.s001.tif]

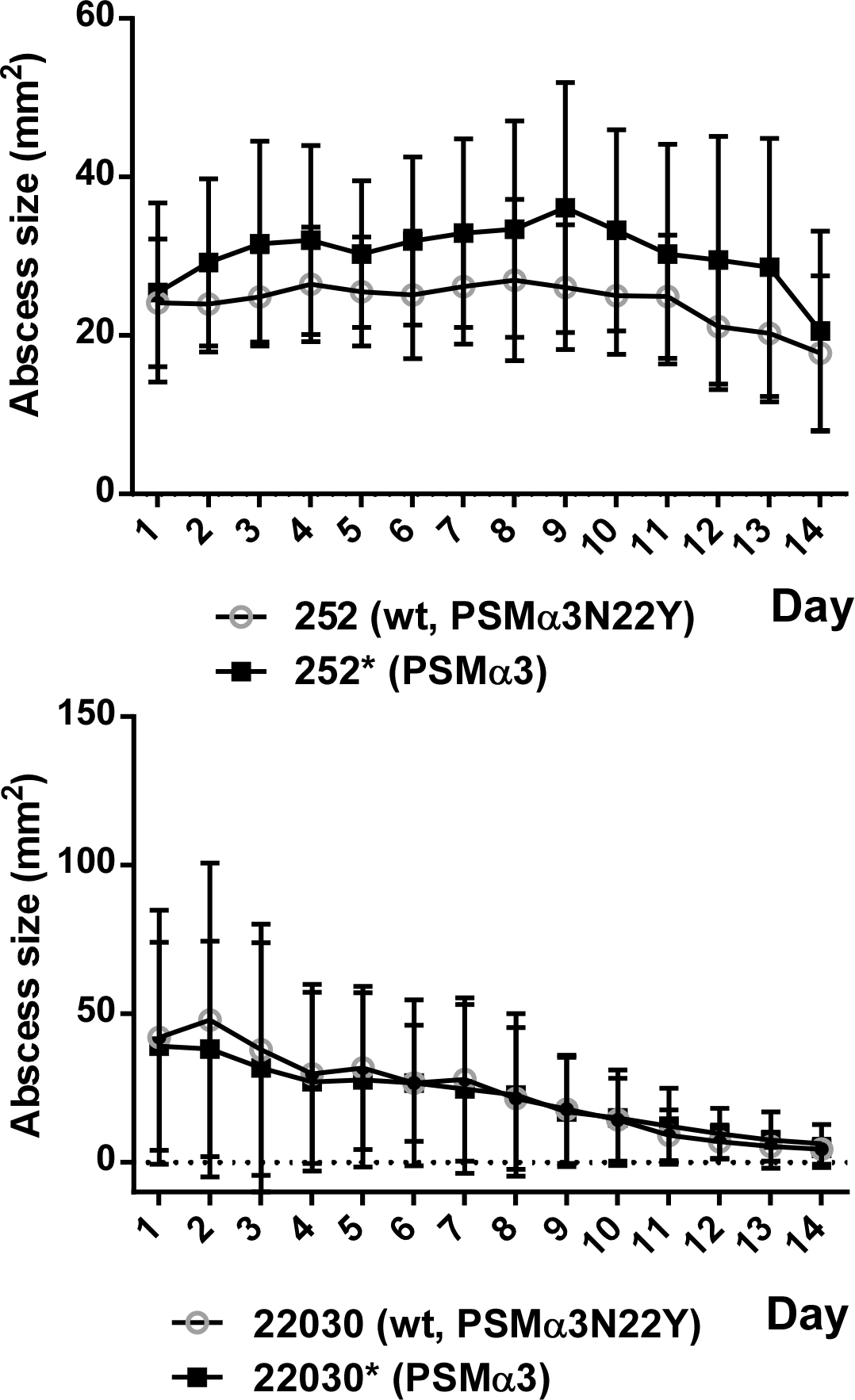

Supplement: Figure S2 — Mouse model of skin infection. Female Crl∶SKH1-hrBR hairless mice were injected subcutaneously with ∼4×10^7^ CFU of S. aureus strains 252 or 252* (number of mice, 25 per group), or ∼5×10^6^ CFU of S. aureus strains 22030 or 22030* (number of mice, 15 per group) in 50 µl of PBS in the left flank of the mouse. The length (L) and width (W) of the abscess or lesion caused by the bacterial infection was measured with an electronic caliper daily for 14 d post infection and calculated using the formula L×W. Typically, strain MRSA 252 caused closed abscesses and strain 22030 open lesions. (TIF) [file ppat.1004298.s002.tif]
